# Hospital Progress and Finance

**Published:** 1923-03

**Authors:** 


### HOSPITAL PROGRESS AND FINANCE.

### THE MAUDSLEY HOSPITAL.

OF the many imperfections in our methods of treating mental disorders none has been more tragic than our dealings with incipient insanity. That there should have been no ready means of watching and curing unhappy people who find themselves on the mental borderland has been a heavy reproach. To certify such persons and place them in asylums has, only too often, been to deprive them of the hope of regaining stability. The splendid Maudsley Hospital, just opened, of which we give an account in our issue this month, is designed to provide the very opportunity that has hitherto been lacking. It will receive no patient save by his own will and at his own desire; all suggestion of the asylum or of physical duress is absent, and its place is taken by brightness and cheerfulness, tact and tenderness. That the Maudsley institution will save many a man and woman from intellectual death is certain, and no gratitude, however vast, can reach the sum of that which is due to the late Dr. Maudsley for his humane and far-sighted foundation. There is room for many more such hospitals, which are unquestionably called materially to reduce the number of people who have hitherto been certified because it was difficult, and perhaps impossible, to do anything else. To save a mind is as signal a work of mercy as to save a body.

### VOLUNTARY HOSPITALS IN NORTH WALES.

THE first annual report of the Voluntary Hospitals Committee for North Wales has now been issued. It concerns the five large hospitals and the sixteen Cottage Hospitals in the six northern counties of the Principality. It appears that eight institutions applied for grants. The Committee recommended grants amounting to £873, and the Commission awarded £709 conditionally. In addition, two other hospitals applied, but since, at the end of 1921, both had an excess of income, the consideration of their needs was deferred. The expenses of the Local Committee, comprising the hire of a room, stationery, postage, and the Secretary's travelling expenses, are estimated at £60, to which each county has been invited to contribute £10. Four have done so, and it is hoped that the remaining two will follow. This first report is interesting because it shows that the work was necessarily limited at first to the recommendation of grants. But the programme now will expand.

### THE "NEW MOVEMENT" IN SHEFFIELD.

IN connection with the social evenings that are proving so useful educationally to the supporters of the hospitals in Marylebone, we may note a scheme on somewhat similar lines now arranged in Sheffield. There the Joint Hospitals' Council, of which Mr. S. R. Lamb is the secretary, has been holding a series of Health Lectures, to encourage the army of regular contributors to realise the benefits that they secure from the hospitals even when they do not need their treatment. The lectures are free, and prominent speakers have discussed, sometimes with the help of illustrations, such subjects as recent scientific discoveries in relation to public health, milk, sleep and sunshine as factors in well-being, and so on. Unlike the Marylebone plan, however, these lectures and demonstrations, which are or will be held in the suburbs as well as in the city, do not take place in the hospitals but in public halls. We suggest that the details of the London plan, which Major Ainsworth Davies explained recently in our columns, should be studied by the authors of the Sheffield plan. The great advantage of the Metropolitan scheme is the human and social side which it imports into what sometimes threatens to be regarded as a mere agency for collecting money.

### CONTRIBUTION SCHEMES AND APPROVED SOCIETIES.

AT the annual meeting of the National Federation of Employees' Approved Societies, Lord Hambleden referred to the 3d. a week scheme of the Hospital Saving Association, of which he is chairman. After declaring that "great appeals were unsatisfactory from every point of view," he said that the desire was to co-ordinate agencies and make great appeals unnecessary. Under the scheme people of limited income could save regularly for the hospitals in their clubs, offices, and social organisations; the sums could be pooled and the hospitals, thus reimbursed, relieve patients from payments. Mr. F. W. Eaton (Underground Railways) criticised the amount of the contribution, which he said was too high, as he had proved to the authorities of St. Thomas's Hospital. Lord Hambleden replied that if it were found to be too much, the sum could easily be reduced. He also explained that large waiting lists made it very difficult to give guarantees for admission.

### COTTAGE HOSPITALS AND CO-OPERATION.

AT a recent meeting of the Voluntary Hospitals Committee for Leicestershire and Rutland, Mr. J. Bond, F.R.C.S., outlined a scheme for a central hospital bureau for the area, by means of which co-operation in regard to the admission and discharge of patients could be secured. It would greatly relieve the central institution if the after-treatment of patients could be arranged in cottage hospitals of the patients' own districts. If each such hospital had a small but efficient out-patients' clinic, where dressings and massage could be undertaken by medical officers, much expense would be saved. The Leicester Royal Infirmary needed a convalescing hospital, not a convalescent home, for patients who still required skilled nursing. This need might be met if each cottage hospital would provide such nursing and medical supervision, provided there were general goodwill and a general desire to co-ordinate all the hospitals in the area. The Leicester Royal Infirmary is still short of beds, and though, we understand, the new wing will be open in the summer, this will not solve the difficulty.

